# Do bleaching gels affect the stability of the masking and caries-arresting effects of caries infiltration—in vitro

**DOI:** 10.1007/s00784-020-03732-4

**Published:** 2020-12-14

**Authors:** Ellen Elisabeth Jansen, Hendrik Meyer-Lueckel, Marcella Esteves-Oliveira, Richard Johannes Wierichs

**Affiliations:** 1 Clinic for Operative Dentistry, Periodontology and Preventive Dentistry, RWTH Aachen University, Pauwelsstraße 30, 52074 Aachen, Germany; 2 Department of Restorative, Preventive and Pediatric Dentistry, University of Bern, Freiburgstrasse 7, 3010 Bern, Switzerland; 3 Department of Cariology, Endodontology and Periodontology, University of Leipzig, Liebigstraße 12, 04103 Leipzig, Germany; 4 Department of Biohybrid & Medical Textiles, Institute of Applied Medical Engineering, RWTH Aachen University, Forckenbeckstraße 55, 52074 Aachen, Germany

**Keywords:** Bleaching, Demineralization, Aesthetics, Enamel, White spot lesions, Staining, Caries infiltration, In vitro

## Abstract

**Objectives:**

The aim of this study was to evaluate the influence of different bleaching gels on the masking and caries-arresting effects of infiltrated and non-infiltrated stained artificial enamel caries lesions.

**Materials and methods:**

Bovine enamel specimens (*n* = 240) with each two sound areas (SI and SC) and each two lesions (DI and DC) were infiltrated (DI and SI), stained (1:1 red wine-coffee mixture,70 days), and randomly distributed in six groups to be bleached with the following materials: 6%HP (HP-6), 16%CP (CP-16), 35%HP (HP-35), 40%HP (HP-40), and no bleaching (NBl,NBl-NBr). Subsequently, specimens were pH-cycled (28 days, 6 × 60 min demineralization/day) and all groups except NBl-NBr were brushed with toothpaste slurry (1.100 ppm, 2×/day, 10 s). Differences in colorimetric values (Δ*L*, Δ*E*) and integrated mineral loss (ΔΔ*Z*) between baseline, infiltration, staining, bleaching, and pH cycling were calculated using photographic and transversal microradiographic images.

**Results:**

At baseline, significant visible color differences between DI and SC were observed (Δ*E*_baseline_ = 12.2; *p* < 0.001; ANCOVA). After infiltration, these differences decreased significantly (Δ*E*_infiltration_ = 3.8; *p* < 0.001). Staining decreased and bleaching increased Δ*L* values significantly (*p* ≤ 0.001). No significant difference in ΔΔ*E* was observed between before staining and after bleaching (Δ*E*_bleaching_ = 4.3; *p* = 0.308) and between the bleaching agents (*p* = 1.000; ANCOVA). pH-cycling did not affect colorimetric values (Δ*E*_pH-cycling_ = 4.0; *p* = 1.000). For DI, no significant change in Δ*Z* during in vitro period was observed (*p* ≥ 0.063; paired *t* test).

**Conclusions:**

Under the conditions chosen, the tested materials could satisfactorily bleach infiltrated and non-infiltrated stained enamel. Furthermore, bleaching did not affect the caries-arresting effect of the infiltration.

**Clinical relevance:**

The present study indicates that bleaching is a viable way to satisfactorily recover the appearance of discolored sound enamel and infiltrated lesions.

## Introduction

In recent years, the aesthetic demands for brighter and whiter teeth by patients have increased [1]. As a consequence, dentists often have to deal with extrinsic or intrinsic staining as well as non-cavitated initial lesions—also known as white spot lesions (WSL). The latter frequently being associated with fixed orthodontic treatments [2]. After orthodontic treatments, a WSL prevalence up to 97% has been reported [2]. Successful masking of a WSL by remineralizing regimes only seems to be related to the severity of lesion. When being slightly visible, initial WSL can often be completely remineralized naturally since fixed elements—increasing the plaque retention—have been removed. By the additional use of fluorides, e.g., in form of fluoride varnish remineralization can be increased [3]. However, clearly visible from a social distance, more severe WSL cannot be visually masked naturally and by fluoride alone, but remain visible for life. Thus, for more severe WSL, micro-invasive treatments are indicated.

Resin infiltration technique is one method to mask initial non-cavitated lesions. After removing the less porous pseudointact surface layer of the caries lesion by using HCl, the lesion microporosities are penetrated with a low-viscosity resin [4]. Due to the similar refractive indices (RI) of the resin infiltrant (RI of infiltrant 1.52) compared with apatite (RI = 1.62), light scattering is, thus, reduced and visual color differences to enamel are decreased directly after application. The masking effect has been reported in vitro [4, 5] as well as in vivo [6, 7]. However, in vitro, it could also be observed that after subsequent staining color differences of infiltrated lesions increased relatively when compared to non-infiltrated demineralized or sound surfaces [4]. In a second study, a higher staining susceptibility was observed for infiltrated surfaces when compared to different bonding agents [8]. Although the pre-demineralization procedure of the lesions and the application procedure of the tested agent were not described in the second study, the results of both studies indicated that infiltrated lesions may discolor over time. This, of course, raises the question if the appearance of discolored infiltrated lesions can be recovered or not.

Bleaching of teeth has been shown to be a conservative aesthetic solution with regard to discoloration [9]. Hydrogen peroxide (HP) and carbamide peroxide (CP) have both been used as bleaching agents [9]. Depending on the bleaching technique, concentrations vary between 6 and 40% [10]. Generally, lower concentrations of CP are used for at-home bleaching whereas higher concentrations of both CP and HP are used for in-office bleaching. Although at-home bleaching was preferred by the patients compared with in-office bleaching [11], both techniques can significantly brighten the teeth with extrinsic discoloration [10]. However, until now, it remains unclear if infiltrated non-cavitated initial lesions can be bleached successfully. Only two studies analyzed the bleaching effects on infiltrated non-cavitated initial lesions—one using 16%CP for at-home bleaching technique [12] and one using 35%HP in-office bleaching technique [13]. In both studies, no significant difference in the CIE L*a*b* values were observed between infiltrated lesions and sound enamel. However, only one of the two studies analyzed if stained infiltrated lesions can successfully be bleached but the depths of the infiltrated lesions were not reported. Furthermore, none of the studies analyzed if the caries-arresting effect of the infiltrant is affected by bleaching.

The influence of bleaching materials on composite materials is discussed controversially [14–17]. This is based on a large number of different bleaching and composite materials. An increased surface porosity with crack formation in different composite materials after bleaching was observed in one of the cited studies [14]. In another study, this effect could only be observed with highly concentrated bleaching agents (35% CP), while bleaching agents with a lower concentration (10% CP) did not negatively affect the surface [15]. Further studies indicated, if bleaching materials are used correctly, either high or low-concentration, bleaches will damage the surface structure [16, 17]. However, if increased surface porosities or cracks can affect the caries-arresting effect of the infiltration remains unclear until now. Thus, the aim of this in vitro study was to evaluate the influence of different hydrogen peroxide (HP) and carbamide peroxide (CP) bleaching gels on the masking effect (primary aim) and the caries-arresting effect (secondary aim) of infiltrated and non-infiltrated stained artificial enamel caries lesions. The null hypotheses were that, firstly, no significant difference in the bleaching effect can be observed between the tested bleaching agents but for all compared with the negative control (no bleaching) and that, secondly, bleaching does not affect the masking and caries-arresting effects of the infiltration.

## Materials and methods

### Specimen preparation

**Fig. 1:**
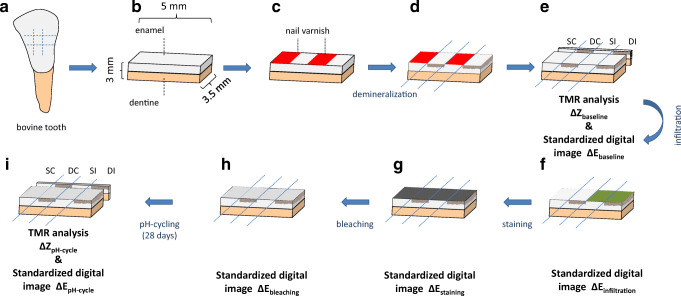
Specimen preparation. (a) Frontal view of a bovine incisor and lines for cutting perpendicular and parallel to the long axis of the tooth crown; (b) Prepared specimens (5 mm × 3 mm × 3.5 mm); (c) Specimen covered with acid resistant nail varnish; (d) Pre-demineralized specimen; (e) Preparation of the 100-μm slices for baseline TMR analysis (*s*ound/positive *c*ontrol area (SC), *d*emineralized *i*nfiltrated area (DI); *s*ound *i*nfiltrated area (SI) and *d*emineralized/negative *c*ontrol area (DC)); (f) Infiltrated specimen (green, only DI and SI); (g) Stained specimen (black); (h) Bleached specimen (gray); (i) Preparation of the 100-μm slices for TMR analysis after pH-cycling

Bovine incisors were extracted from freshly slaughtered cattle (negative BSE test), cleaned, and preserved in 0.08% thymol. The teeth were separated in 400 enamel blocks (5 mm × 3.5 mm × 3 mm) using a diamond band saw (Exakt 300; Exakt Apparatebau, Norderstedt, Germany) [18] under constant water irrigation. The enamel blocks were embedded in epoxy resin (Technovit 4071; Heraeus Kulzer, Hanau, Germany), ground flat, and polished (4000 grit; silicon carbide, Phoenix Alpha, Wirtz-Buehler, Düsseldorf, Germany; Mikroschleifsystem Exakt, Exakt Apparatebau, Norderstedt, Germany). Subsequently, a commercial red acid-resistant varnish was applied to cover two windows of the enamel surface of each specimen (*s*ound/positive *c*ontrol area (SC) and *s*ound *i*nfiltrated area (SI)) (Fig. 1).

### Baseline

To create artificial enamel caries lesions with pseudo-intact surface in the uncovered areas (*d*emineralized/negative *c*ontrol area (DC) and *d*emineralized *i*nfiltrated area (DI)), specimens were stored in a demineralization solution (2.5 ml solution/mm^2^ enamel surface) [19]. The solution contained 50 mM acetic acid, 3 mM CaCl_2_, 3 mM KH_2_PO_4_, 6 μM methylhydroxydiphosphonate, and traces of thymol (pH 4.95; 37 °C). During that period, the pH was monitored daily. If necessary, it was adjusted with small amounts of either 10% HCl or 10 M KOH to maintain a constant pH value. The nail polish was then carefully removed using cotton pellets and acetone. The other surfaces were protected with a liquid rubber dam. At the end, 240 specimens with a mean (95% confidence interval (CI)) baseline mineral loss (Δ*Z*_baseline, DI_) of 4067 (3771; 4361) vol% × μm and lesion depth (LD_baseline, Dl_) of 171.2 (163.2; 178.8) μm were chosen from the 400 specimens originally prepared [20]. The respective colorimetric values for surface DI were 76.8 (76.2; 77.5) for L*, − 4.6 (− 4.8; − 4.4) for a* and − 3.5 (− 3.8; − 3.2) for b*.

### Power calculation

The number of specimens per group was calculated on the basis of a previous study [5]. The *α*-error was set at 5%. Since for the retrospective power analysis with 38 specimens per group has still provided a power of at least 100% for the comparison between time points Δ*E*_baseline_ and Δ*E*_infiltration_, the comparison between Δ*E*_baseline_ and Δ*E*_pH-cycle_ and the comparison between Δ*E*_baseline_ and Δ*E*_pH-cycle_, no additional specimens were involved in the study.

### Infiltration

Icon® (DMG, Hamburg, Germany) was applied according to the manufacturer’s instructions, and only the etching procedure was adapted to the bovine teeth. For 5 s, DI and SI were etched using 37% phosphoric acid gel (Total Etch; Vivadent, Schaan, Liechtenstein) (instead 120 s using HCl as instructed) [4, 21]. Subsequently, the acid gel was removed with distilled water for 30 s. Then, the surface was dried using Icon Dry® for 30 s and infiltrated using Icon Infiltrant® for 3 min. Subsequently, excess material was carefully removed by means of cotton pellets, and the resin was light-cured for 40 s. The application of Icon Infiltrant® was repeated (1 min) and light-cured again (40 s) [5]. After the application the treated surface was polished as recommended by the manufacturer. For this, a polishing cup (Pro-Cup Soft-Light Blue, Kerr, Biberach, Germany) was used.

### Staining

The specimens were stored in a remineralization solution for 70 days [4, 8, 22]. During this period once daily for 10 min, all specimens were immersed once daily for 10 min in a 1:1 mixture of red wine (Flamand Rouge, Edeka, Hamburg, Germany) and coffee infusion (25 g of powder to 250 mL of water; Nescafe Gold, Nestlé, Frankfurt, Germany) Thus, extrinsic dietary staining was simulated.

### Bleaching

The specimens were randomly divided into 6 groups each. Over a period of 10 days before the pH-cycling, specimens were treated with the respective bleaching materials:

- *n*o *bl*eaching, *n*o *br*ushing (NBl-NBr)
- *n*o *bl*eaching, but brushing (NBl)
- 6% *h*ydrogen *p*eroxide bleaching gel for at-home bleaching technique (pH 4.6–6.6; Opalescence Go 6%, Ultradent Products, South Jordan, Utah, USA) and brushing (HP-6)
- 16% *c*arbamide *p*eroxide gel for at-home bleaching technique (pH 6.0–7.2; Opalescence PF 16%, Ultradent Products) and brushing (CP-16)
- 35% *h*ydrogen *p*eroxide bleaching gel for in-office bleaching technique (pH 5.6–6.9 [23]; HP AutoMixx 35%, FGM, Joinville, SC, Brazil) and brushing (HP-35)
- 40% *h*ydrogen *p*eroxide bleaching gel for in-office bleaching technique (pH 6.0–8.5; Opalescence Extra Boost, Ultradent Products) and brushing (HP-40)

Specimens of NBl-NBr and NBl were stored in distilled water all the time.

Specimens of HP-6 and CP-16 were covered with a layer of approximate 1 mm of the bleaching gel for 8 h in a humid atmosphere at 37 °C once daily. Furthermore, the specimens were stored in distilled water in the remaining time (37 °C) [24]. To not affect the results by differing application times, the specimens in both groups were bleached according to the same protocol [24]. Specimens of HP-40 and HP-35 were treated twice on two days (day 1 and day 5) at 7-h intervals. For this purpose, the specimens were covered with a layer of 1 mm of bleaching gels for 30 min at room temperature. In the time between the treatments, the specimens were stored in distilled water (37 °C) [24]. To not affect the results by differing application times, the specimens in both groups were bleached according to the same protocol [24].

Prior to bleaching, the surfaces were dried with cotton wool pellets. After treatment and before retransferring to the distilled water, the bleaching agents were carefully removed by rinsing with distilled water for 30 s.

### pH-cycling

pH-cycling started right after bleaching. For this, a computer-controlled pH-cycling and brushing machine [5] was used to simulate oral pH-fluctuation patterns and daily tooth brushing for 28 days. The pH-cycling involved 6 demineralization periods of 1 h each (total 6 h/day) and 6 remineralization periods of at least 2 h during the day and a longer overnight period (total 18 h/day). The remineralization solution contained 1.5 mM CaCl_2_, 0.9 mM KH_2_PO_4_, and 20 mM N-2-hydroxyethylpiperazine-N’2-ethanesulfonic acid (HEPES) as buffer, pH 7.0 (37 °C). The demineralization solution contained 6 μM methylhydroxydiphosphonate, 3 mM CaCl_2_, 3 mM KH_2_PO_4_, and 50 mM acetic acid adjusted to pH 4.87 (37 °C) [19]. The pH-cycling solutions were refreshed with every cycle (6×/day). The amounts of each solution were large enough to prevent the solutions from becoming saturated with or depleted of mineral ions (0.7 ml solution/mm^2^ enamel surface). Before the first and last remineralizing phase, specimens of all groups (except NBl-NBr) were brushed for 10 s (Oral-B Indicator; Proctor & Gamble, Schwalbach am Taunus, Germany) with a fluoride dentifrice slurry (1100 ppm F^-^ as NaF; Crest Cavity Protection, Proctor & Gamble, Schwalbach am Taunus, Germany). Dentifrice slurries were prepared with deionized water in a ratio of 1:2 (toothpaste:water) parts by weight and refreshed every two days [25]. The dentifrice slurry remained on each specimen for another 110 s. Subsequently, the specimens were perfused with distilled water to remove the slurry. In total, brushing procedure for each specimen lasted 120 s, hence simulating the recommended brushing time of 2 min. Specimens of group NBl-NBr were not brushed at any time. The machine was adjusted to a constant brushing frequency of 60 strokes/min and a constant brushing load of 1.5 N [26].

### Colorimetric analysis

Stability, accuracy, and reliability of colorimetric measurements were evaluated as described previously [4, 5]. Within a lightproof black box and under standardized conditions (aperture of f45, shutter speed at 1/60 s, and image ISO sensitivity 200), digital photographs of moist specimens were obtained using a fixed SLR camera (Nikon D7000, Nikon, Tokyo, Japan). Images of the specimens with a black background were taken with a macro-lens (Nikon 105 mm, 1:2.8, Nikon) and a ring flash (Sigma Em-140 DG, Nikon, lightening intensity 1/4, reloaded batteries) at the following instants of times: after pre-demineralization (Δ*E*_baseline_), after infiltration (Δ*E*_infiltration_), after staining (Δ*E*_staining_), after bleaching (Δ*E*_bleaching_), and after pH-cycling (Δ*E*_pH-cycle_). In order to be able to control the standardized conditions, with each specimen, a gray card (Mennon Gray Cards Neutraal 18 %; Mennon USA, USA) was photographed.

Evaluation of digital images (RAW-format) was performed using Photoshop CS6 extended (Adobe, San Jose, USA) [5]. For all timepoints, the same area of each specimen was evaluated. The CIE-L*a*b* color system was used to analyze optical results. It records colorimetric parameters three-dimensionally: lightness (L*; 0 to + 100), green-red chromaticity (a*; − 150 to + 100), and blue-yellow chromaticity (b*; − 100 to + 150). Within a window of 105 × 105 picture elements, the CIE L*a*b* values were measured. For the chosen 240, the colorimetric values for the surfaces DI, DC, SI, and SC at baseline were as follows: 76.8 (76.2; 77.5) for L*_DI_, − 4.6 (− 4.8; − 4.4) for a*_DI_ and − 3.5 (− 3.8; − 3.2) for b*_DI_; 79.5 (79.0; 80.0) for L*_DC_, − 3.9 (− 4.1; − 3.7) for a*_DC_ and − 1.9 (− 2.2; − 1.6) for b*_DC_; 69.3 (68.8; 69.9) for L*_SI_, − 1.5 (− 1.7; − 1.3) for a*_SI_ and 2.4 (− 2.0; − 2.9) for b*_SI_; 66.3 (66.7; 66.8) for L*_SC_, − 1.2 (− 1.4; − 1.0) for a*_SC_, and 0.9 (− 0.5; − 1.2) for b*_SC_.

Color differences (Δ*E*) and variation in lightness (Δ*L*) were calculated (Excel 2010, Microsoft, Redwood, USA) using the equation Δ*E* = ((*L*_sound*_ − *L*_caries*_)^2^ + (*b*_sound*_ − b_caries*_)^2^ + (*a*_sound*_ − *a*_caries*_)^2^)^1/2^ and Δ*L* = *L*_sound*_ − *L*_caries*_, respectively. Changes in colorimetric values (e.g., ΔΔ*E*_3_ = Δ*E*_baseline_ − Δ*E*_bleaching_) were calculated.

### Transversal microradiographic analysis

After pre-demineralization and after pH-cycling, thin plane-parallel sections were prepared. Changes in mineral loss (ΔΔ*Z* = Δ*Z*_baseline_ − Δ*Z*_pH-cycle_) were calculated using transversal microradiographic images. Microradiographs of the enamel specimens were obtained and analyzed as described previously [27, 28].

### Statistical analysis

Data were analyzed using SPSS statistical software (SPSS 25.0; SPSS, Munich, Germany). Variables were tested for normal distribution (Shapiro-Wilk test). Within one group, changes in mineral loss after pre-demineralization (Δ*Z*_baseline_/LD_baseline_) and after pH-cycling (Δ*Z*_pH-cycle_/LD_pH-cycle_) were analyzed using two-tailed paired *t* tests. Changes in colorimetric values after pre-demineralization (Δ*E*_baseline_), after infiltration (Δ*E*_infiltration_), after staining (Δ*E*_staining_), after bleaching (Δ*E*_bleaching_), and after pH-cycling (Δ*E*_pH-cycle_) within one group were analyzed using analysis of covariance (ANCOVA) and Bonferroni post hoc tests.

Analysis of covariance (ANCOVA) for sound and demineralized surfaces and Bonferroni post hoc tests were also used for pair-wise multiple-comparisons to detect differences in changes of mineral loss (∆∆*Z*) and colorimetric values (ΔΔ*E*). More technically the ANCOVA statistical model may be described as a general linear mixed model with transversal microradiographic (TMR)/colorimetric data and treatment as fixed effects. All tests were performed at a 5% level of significance.

## Results

### Colorimetric analysis

**Fig. 2:**
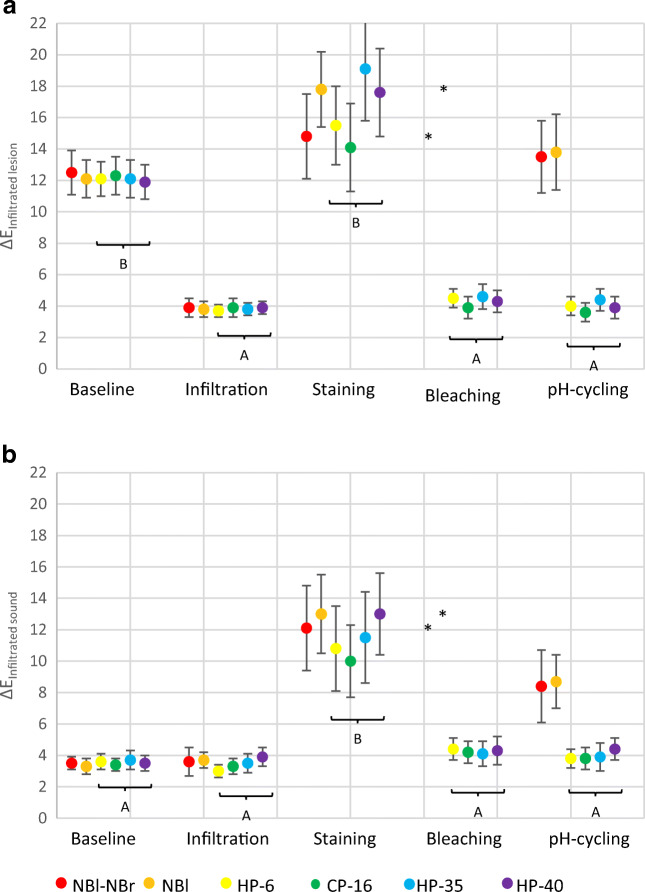
Means with confidence intervals (95%) of colorimetric values (Δ*E*) for demineralized specimens at different time points. Colorimetric values of infiltrated demineralized enamel can be seen in **a** and colorimetric values of infiltrated sound enamel can be seen in **b**. Different letters indicate significant differences between different time points (*p* < 0.05; Bonferroni post hoc test). Asterisk symbol indicates that NBl-NBr and NB were not bleached; thus, no values for Δ*E*_bleaching_ are presented

**Fig. 3:**
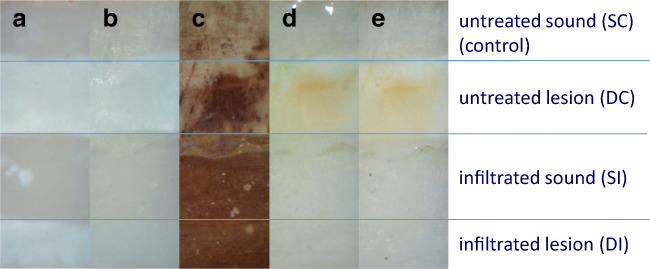
Photographs of one representative demineralized specimen at different times of treatment: Baseline (a), Infiltration (b), Staining (c), Bleaching (d), and pH-cycle (e). Staining discolored all areas considerably. Bleaching recovered the appearance of discolored sound enamel and infiltrated lesions. After bleaching, infiltrated lesion was visually not discriminable from sound enamel. The results remained stable during pH-cycling

At baseline, significant visible color differences between DI and SC were observed within each group (Δ*E*_baseline_ = 12.2; *p* < 0.001; ANCOVA, Table 1, Fig. 2). After infiltration, these differences decreased significantly (Fig. 3), (Δ*E*_infiltration_ = 3.8; *p* < 0.001; ANCOVA).

**Table 1:** Means with confidence intervals (95%) of the CIE L*a*b* values for demineralized specimens

		NBl-NBr			NBl			HP-6			CP-16			HP-35			HP-40		
*N*^#^		36			38			39			39			38			39		
Infiltrated lesion	Δ*E*_baseline_	12.5 (11.1;13.8)	*A*	*a*	12.1 (10.9;13.3)	*A*	*a*	12.1 (11;13.3)	*A*	*a*	12.3 (11.1;13.4)	*A*	*a*	12.1 (10.9;13.3)	*A*	*a*	11.9 (10.8;13)	*A*	*a*
	Δ*E*_infiltration_	3.9 (3.3;4.4)	*A*	*b*	3.8 (3.3;4.3)	*A*	*b*	3.7 (3.3;4.2)	*A*	*b*	3.9 (3.3;4.4)	*A*	*b*	3.8 (3.4;4.3)	*A*	*b*	3.9 (3.5;4.3)	*A*	*b*
	Δ*E*_staining_	14.8 (12.1;17.4)	*A*	*a*	17.8 (15.4;20.1)	*A*	*a*	15.5 (13;18)	*A*	*c*	14.1 (11.3;16.8)	*A*	*a*	19.1 (15.8;22.3)	*A*	*c*	17.6 (14.8;20.4)	*A*	*c*
	Δ*E*_bleaching_	*			*			4.5 (3.9;5)	*A*	*a*	3.9 (3.2;4.5)	*A*	*b*	4.6 (3.8;5.4)	*A*	*b*	4.3 (3.6;4.9)	*A*	*b*
	Δ*E*_pH-cycle_	13.5 (11.2;15.8)	*A*	*a*	13.8 (11.4;16.3)	*A*	*a*	4 (3.4;4.6)	*B*	*a*	3.6 (3;4.1)	*B*	*b*	4.4 (3.7;5.1)	*B*	*b*	3.9 (3.2;4.7)	*B*	*b*
Infiltrated sound	Δ*E*_baseline_	3.5 (3.1;4)	*A*	*a*	3.3 (2.8;3.8)	*A*	*a*	3.6 (3.1;4)	*A*	*a*	3.4 (3;3.9)	*A*	*ab*	3.7 (3.1;4.2)	*A*	*a*	3.5 (3;4.1)	*A*	*a*
	Δ*E*_infiltration_	3.6 (2.7;4.6)	*A*	*a*	3.7 (3.2;4.3)	*A*	*a*	3 (2.6;3.3)	*A*	*a*	3.3 (2.8;3.8)	*A*	*a*	3.5 (2.9;4)	*A*	*a*	3.9 (3.3;4.6)	*A*	*a*
	Δ*E*_staining_	12.1 (9.4;14.7)	*A*	*c*	13 (10.5;15.5)	*A*	*c*	10.8 (8.1;13.4)	*A*	*b*	10 (7.7;12.3)	*A*	*c*	11.5 (8.6;14.5)	*A*	*b*	13 (10.4;15.5)	*A*	*b*
	Δ*E*_bleaching_	*			*			4.4 (3.7;5)	*A*	*a*	4.2 (3.5;4.9)	*A*	*a*	4.1 (3.3;4.9)	*A*	*a*	4.3 (3.4;5.2)	*A*	*a*
	Δ*E*_pH-cycle_	8.4 (6.1;10.8)	*A*	*b*	8.7 (7;10.5)	*A*	*b*	3.8 (3.2;4.4)	*B*	*a*	3.8 (3.1;4.4)	*B*	*ab*	3.9 (3;4.8)	*B*	*a*	4.4 (3.7;5.1)	*B*	*a*
Untreated lesion	Δ*E*_baseline_	14 (12.9;15.1)	*A*	*a*	13.4 (12.2;14.5)	*A*	*a*	13.6 (12.5;14.8)	*A*	*a*	13.6 (12.5;14.8)	*A*	*a*	13.8 (12.7;15)	*A*	*a*	13.4 (12.4;14.5)	*A*	*a*
	Δ*E*_infiltration_	13.2 (12.1;14.2)	*A*	*a*	13.4 (12.3;14.6)	*A*	*a*	14.1 (13;15.1)	*A*	*a*	13.5 (12.4;14.5)	*A*	*a*	12.8 (11.7;13.9)	*A*	*a*	12.1 (11.3;12.9)	*A*	*a*
	Δ*E*_staining_	15.8 (12.8;18.9)	*A*	*a*	13.1 (10.7;15.5)	*A*	*a*	14.4 (12.2;16.7)	*A*	*a*	14.9 (12.6;17.1)	*A*	*a*	13.6 (12;15.3)	*A*	*a*	20 (16.3;23.7)	*B*	*c*
	Δ*E*_bleaching_	*			*			10.6 (9.3;11.9)	*A*	*b*	10.5 (9.3;11.6)	*A*	*b*	14.8 (12.9;16.7)	*B*	*a*	9.3 (8.1;10.5)	*A*	*b*
	Δ*E*_pH-cycle_	17.7 (14;21.3)	*A*	*a*	12.4 (10.2;14.6)	*B*	*a*	9.8 (8.5;11)	*B*	*b*	10.8 (9.6;12)	*B*	*b*	13.5 (11.8;15.1)	*B*	*a*	10 (8.9;11.1)	*B*	*b*

Means with confidence intervals (95%) of the CIE L*a*b* values for specimens after pre-demineralization (Δ*E*_baseline_), infiltration (Δ*E*_infiltration_), staining (Δ*E*_staining_), bleaching (Δ*E*_bleaching_), and pH-cycling (Δ*E*_pH-cycle_). Different letters indicate significant differences between the groups within one time point (large caps), (*p* < 0.05; Bonferroni post hoc test) as well as within one group between different time points (small caps) (*p* < 0.05; Bonferroni post hoc test)

*NBl-NBr and NB were not bleached; thus, no values for Δ*E*_bleaching_ are presented

^#^If specimens were too dry or too wet to be analyzed, they were excluded. Thus, the number of analyzed specimens per group slightly varies

Staining decreased and bleaching increased Δ*L* values significantly (*p* < 0.001). For HP-6, CP-16, HP-35, and HP-40, no significant difference in colorimetric values could be observed between before staining and after bleaching (Δ*E*_bleaching_ = 4.3; *p* = 0.3080). Furthermore, no significant difference between the bleaching agents was observed (*p* = 1.000). However, after bleaching, DC was significantly brighter than DI (*p* ≤ 0.001). pH-cycling did not affect colorimetric values (Δ*E*_pH-cycling_ = 4.0; *p* = 1.000).

### Transversal microradiographic analysis

For infiltrated demineralized enamel, no significant change in mineral loss between values after initial demineralization (Δ*Z*_baseline,DI_ (95% CI): 4067 (3771; 4361) vol% × μm) and after pH-cycling (Δ*Z*_pH-cycle,DI_ (95% CI): 3842 (3573;4103) vol% × μm) was observed (*p* ≥ 0.063; paired t test). For non-infiltrated demineralized enamel (Δ*Z*_baseline,DC_ (95% CI): 4588 (4279; 4895) vol% × μm; Δ*Z*_pH-cycle,DC_ (95% CI): 5223 (4882; 5564) vol% × μm), a significant increase in mineral loss was observed for NBl-NBr as well as for acidic bleaching agents (HP-6r and HP-35) (*p* ≤ 0.037, Table 2), whereas no significant increase could be observed for NBl and neutral bleaching agents (CP-16an HP-40) (*p* ≥ 0.184). Furthermore, no significant difference between the groups could be observed for ΔΔ*Z*_DC_ (*p* ≥ 0.438, ANCOVA, Table 2) and ΔΔ*Z*_DI_ (*p* = 1.000). However, ΔΔ*Z*_DC_ was significantly higher compared with ΔΔ*Z*_DI_ (*p* < 0.001, paired *t* test).

**Table 2:** Mean (95% confidence interval) mineral losses after initial demineralization (Δ*Z*_baseline,DI_/Δ*Z*_baseline,DC_), after pH-cycling (Δ*Z*_pH-cycling,DI_/Δ*Z*_pH-cycling,DC_), and changes in mineral loss (ΔΔ*Z*_DI_/ΔΔ*Z*_DC_) of demineralized infiltrated (DI) and non-infiltrated (DC) lesions

**Intervention**	***N***	**Δ*Z***_**baseline,DI**_ **(vol% × μm)**			**Δ*Z***_**pH-cycling,DI**_ **(vol% × μm)**			p*	**ΔΔ*Z***_**DI**_ **(vol% × μm)**		
NBl-NBr	24	3785	(3188;4382)	*A*	3718	(3158;4279)	*A*	0.747	106	(− 354;567)	*A*
NBl	24	4111	(3248;4975)	*A*	3744	(3072;4416)	*A*	0.322	234	(− 527;996)	*A*
HP-6	24	4636	(3785;5487)	*A*	4455	(3686;5223)	*A*	0.725	24	(− 636;685)	*A*
CP-16	22	3779	(3020;4539)	*A*	3678	(2899;4457)	*A*	0.744	102	(− 537;740)	*A*
HP-35	29	4216	(3661;4772)	*A*	3768	(3209;4327)	*A*	0.063	449	(− 27;924)	*A*
HP-40	24	4210	(3289;5131)	*A*	3662	(2879;4445)	*A*	0.125	322	(− 238;883)	*A*
**Intervention**	***N***	**Δ*Z***_**baseline,DC**_ **(vol% × μm)**			**Δ*Z***_**pH-cycling,DC**_ **(vol% × μm)**			p*	**ΔΔ*Z***_**DC**_ **(vol% × μm)**		
NBl-NBr	28	4690	(4012;5367)	*a*	6041	(5219;6863)	*a*	*0.006*	− 1446	(− 2451; − 441)	*a*
NBl	32	4645	(3890;5400)	*a*	5215	(4372;6058)	*a*	0.184	− 605	(− 1480;271)	*a*
HP-6	27	4642	(3863;5421)	*a*	5561	(4654;6467)	*a*	*0.037*	− 771	(− 1702;161)	*a*
CP-16	27	4568	(3701;5435)	*a*	4807	(3932;5682)	*a*	0.620	− 275	(− 1284;734)	*a*
HP-35	31	4223	(3507;4938)	*a*	4999	(4261;5737)	*a*	*0.019*	− 776	(− 1417;− 135)	*a*
HP-40	29	4719	(3746;5691)	*a*	4585	(3590;5579)	*a*	0.801	− 18	(− 1103;1067)	*a*

*Italicized *p* values indicate significant differences in mineral losses before and after pH-cycling (two-tailed paired *t* test). Different letters indicate significant differences between treatments among infiltrated (DI, large caps) and non-infiltrated (DC, small caps) demineralized specimens (*p* < 0.05; Bonferroni post hoc test). Negative ΔΔ*Z* values indicate demineralization, and positive ΔΔ*Z* values indicate remineralization

## Discussion

The present in vitro study compared the bleaching effects of different hydrogen peroxide and carbamide peroxide bleaching gels on infiltrated and non-infiltrated stained caries-like enamel lesions. Without any material-depending differences, all bleaching agents were able to significantly whiten sound and demineralized enamel when compared to the negative controls (no bleaching). The primary hypothesis was, thus, accepted. Furthermore, no significant differences in the colorimetric values and mineral contents before staining and after bleaching and pH-cycling could be observed, confirming the second hypothesis that bleaching does not affect the masking and caries-arresting effects of the infiltration.

In the present study, staining significantly increased Δ*E* values of infiltrated demineralized and infiltrated sound enamel (DI and SI, respectively). Δ*E* values of non-infiltrated demineralized enamel remained relatively stable even though they discolored as much as the rest of the specimens. Furthermore, they showed the highest Δ*E* values before and after staining, although color differences of infiltrated lesions increased relatively when compared to non-infiltrated demineralized or sound enamel. Furthermore, no significant differences among the three areas could be observed after staining. These results are in agreement with previous studies [4, 12, 29]. In the first study, staining provided a significant reduction of Δ*L* values in infiltrated lesions, sound enamel, and in untreated lesions without any differences among the three groups [12]. In the second study, after staining, no significant difference could be observed between infiltrated and non-infiltrated lesions as well as (non-infiltrated) sound enamel [29]. In the third study, no significant difference in Δ*E* values could be observed between infiltrated lesions and infiltrated sound enamel [4]. In that study, it could also be observed that color differences of infiltrated lesions increased relatively when compared to non-infiltrated lesions or sound enamel. Furthermore, in one study, it was observed that after staining, infiltrated lesions showed significantly higher Δ*E* values than lesions which were stored in artificial saliva and treated with sodium fluoride daily for eight days before staining [29]. However, in that study, bovine enamel was etched with HCl for 120 s instead of phosphoric acid gel [21] and it was not reported weather the oxygen inhibition layer after infiltration was removed.

By infiltrating the microporosities of the lesions, they were penetrated with a low-viscosity resin. Although resin-based materials are widely used in aesthetic restorations, they are subjected to color alteration over time. For example, triethylene glycol dimethacrylate (TEGDMA)—the main component of the infiltrant—is presumed to increase water absorption [30, 31] and hinder color stability [32]. Consequently, it might be speculated that, if the resin material is able to absorb water, it is also able to absorb other fluids, resulting in the alteration of color [33]. In recent studies on the color stability of infiltrated lesions, polishing after infiltration significantly increased the resistance to subsequent discolorations [4, 34]. However, the appearance of already discolored sound enamel and infiltrated lesions could not be recovered by polishing alone [29]. No significant difference in Δ*L*- and Δ*E* values before and after polishing could be observed. Interestingly, the appearance of discolored sound enamel and infiltrated lesions could recently be recovered by bleaching—another (non-invasive) aesthetic solution to whiten teeth and to remove discoloration [12]. Although, firstly, bovine enamel were etched with HCl instead of phosphoric acid gel [21] and, secondly, not reporting lesions depths of the lesions, no significant difference in the CIE L*a*b* values were observed between infiltrated demineralized and sound enamel. The results are in agreement with the present study. Bleaching significantly increased *L* values of infiltrated demineralized as well as infiltrated sound enamel. Furthermore, colorimetric differences (Δ*E*) between infiltrated demineralized and infiltrated sound enamel after infiltration and after bleaching did not significantly differ, indicating that the visual appearance could be recovered.

No significant difference in the bleaching effects between the 6%HP-based and 16%CP-based agents for at-home bleaching could be observed. Although this result seems to be consistent with previous in vivo studies [35, 36], a recent meta-analysis on HP and CP for at-home bleaching revealed that CP-based agents showed slightly significantly higher color changes than HP-based ones [10]. Interestingly, the active whitening component in both bleaching agents is the same: HP—*a* 10% CP gel consists of roughly 3.5% HP and 6.5% urea [10]. Furthermore, the concentrations of the active HP were higher in HP-based than in CP-based agents. However, it was speculated that the release of HP in tray-delivered CP-based agents is slower than in HP-based ones [37]. Consequently, HP for oxidation in CP-based agents is available over a longer time than in HP-based agents, resulting in higher color changes [10]. It might, furthermore, be speculated that in the present in vitro study, both bleaching agents (HP and CP) whitened the analyzed surfaces to the maximum. The visual appearance of infiltrated demineralized and sound enamel surfaces was successfully recovered and a further whitening effect of surfaces being infiltrated with a low viscosity resin was not feasible. Consequently, under the present in vitro setting, the presumed slightly higher whitening effect of CP-based agents was not expectable and not representable.

In the previous studies on color stability of infiltrated surfaces, specimens were stained for either 10 [34] or 14 days [12]. Although surfaces significantly discolored within these periods, it was not reported if pigments of red wine-coffee mixtures were deposited as surface precipitates on the enamel surfaces or incorporated into the enamel surfaces. In contrast, in the present study, specimens were stained within 70 days (as performed before [22]) and it could be shown that pigments were not only deposited on the surface but were also incorporated up to a depth of 99 μm in both sound and demineralized non-infiltrated and up to a depth of 47 μm in sound and demineralized infiltrated enamel (data not presented).

A few studies investigated the influence of staining [12, 29, 34] and bleaching [12] on the color stability of infiltrated demineralized and sound enamel. However, the influence of bleaching on the caries-arresting effects of the infiltration has not been analyzed until now. In the present study, no significant change in mineral content for infiltrated surfaces during pH-cycling and no effect of the bleaching gels on the caries inhibition by infiltration could be observed. This is in agreement with previous non-bleaching pH-cycling studies [5, 38]. However, after infiltration, specimens were stored in remineralization solution for 70 days, before they were pH-cycled for 28 days. Consequently, it might be speculated that the present in vitro protocol resulted in relative neutral conditions and that no (significant) changes in mineral loss in infiltrated enamel were observed due to the neutral conditions. Nevertheless, in non-infiltrated demineralized enamel, a significant demineralization could be observed in NBl-NBr (no bleaching as well as no brushing during pH-cycling), whereas in NBl (no bleaching but brushing during pH-cycling), no significant demineralization could be observed, indicating netto-demineralizing conditions during pH-cycling.

In the present study, a significant demineralization could not only be observed in NBl-NBr but also for acidic bleaching agents (HP-6 and HP-35). Contrastingly, no significant increase could be observed for the neutral bleaching agents (CP-16 and HP-40). These results are in agreement with previous studies showing pH-depending demineralization [39], erosively induced demineralization [40] and changes in the surface morphology [41]. In contrast, other studies showed no correlation between the pH of bleaching agents and tooth wear [42], or between pH and enamel demineralization [43, 44]. Interestingly, one study indicated that bleaching agents might induce pH-depending enamel morphology alterations in vitro but that the observed demineralizing effect might be irrelevant in in situ conditions [45]. Since, firstly, the present study did not primarily analyze the influence of the pH of bleaching agents, secondly, the in vitro models as well as the outcomes in the cited studies and the present varied widely and, thirdly, the observation that under clinical conditions the observed effects might be masked, the clinical significance of the bleaching agents’ pH remains unclear.

This study is subject to a number of limitations. First, bovine enamel instead of human enamel was used. Although several studies demonstrated that human and bovine enamel react similarly, slight differences between both substrates were also reported [46]. Second, due to the bovine artificial caries lesions, the etching procedure had to be modified [21]. Thus, an etching procedure with phosphoric acid for 5 s was chosen (instead 120 s using HCl as instructed by the manufacturer), as this protocol was already tested in previous studies using bovine teeth [4, 5, 21]. Third, demineralized lesions with mean lesion depths of 171 μm were used to analyze the bleaching effects. From a clinical point of view, they would probably be difficult to detect in vivo as discussed previously [20]. However, the initiation and progression of enamel caries lesions seems to be extremely slow and anti-caries effects are assumed to be limited to the outer 150–200 μm of the lesion (at deeper levels, no difference was observed compared with placebo). Fourth, a pH-cycling model is a chemical caries model without any biofilm. Thus, no antimicrobial interferences in the staining (or bleaching) could be analyzed. Fifth, for colorimetric analysis the sound non-infiltrated surfaces (SC) served as “intra-specimen” reference to analyze the change in ∆*E* and ∆*L* values. However, these surfaces were stained as well. Thus, to objectively describe the darkening effect of the staining and the whitening effect of the bleaching procedure, the colorimetric analysis had to be modified. For these analyses, the gray card instead of SC was used as specimen reference to calculate Δ*E* and Δ*L* values. Although this modification was also performed previously [4], absolute colorimetric results and measured color differences between different time points should be interpreted with caution.

Under the pH-cycling conditions chosen, the tested bleaching agents could successfully recover the visual appearance of infiltrated and non-infiltrated stained caries-like enamel lesions. Furthermore, the masking and the caries-arresting effects of infiltrated lesions remained stable after bleaching and pH-cycling.

## Data Availability

Data can be requested from the authors.
